# A comparison of clinical efficacy between different surgical approaches for popliteal cyst

**DOI:** 10.1186/s13018-017-0659-z

**Published:** 2017-10-25

**Authors:** Bo Yang, Fengchun Wang, Yanhua Lou, Juan Li, Lei Sun, Lei Gao, Feng Liu

**Affiliations:** Department of Orthopaedics, Tai’an Central Hospital, Tai’an, Shandong 271000 China

**Keywords:** Arthroscopy, Popliteal cyst, Internal drainage, Open excision

## Abstract

**Background:**

A popliteal cyst is a benign swelling with synovial fluid located behind the knee joint. Popliteal cysts are often asymptomatic; however, symptomatic cysts may cause pain and may need surgery interventions. Here, we performed a perspective study to compare the clinical efficacy of different surgical approaches, including traditional open excision and advanced arthroscopic treatment.

**Methods:**

A total of 76 patients with popliteal cysts were assigned into three groups by a randomized complete block design. Group A included 32 patients (15 males and 17 females, age 55.3 ± 9.8 years) who received arthroscopic internal drainage of the cysts. Group B included 19 patients (9 males and 10 females, age 55.4 ± 7.6 years) who received open excision after arthroscopic treatment. Group C included 25 patients (11 males and 14 females, age 54.2 ± 8.5 years) who received open excision. All patients were followed up for an average of 13.7 ± 2.4 months. The following parameters were compared: the time of surgery, during surgery, the length of incision, the incision healing rate, the visual analog scale (VAS) for pain, the hospitalization time, the rate of recovery to level 0–1 cysts, the recurrence rate, and the Lysholm score.

**Results:**

Group A exhibited significant better outcomes compared to groups B and C in the length of incision (1.6 ± 0.1 cm), the incision healing rate (100%), the postoperative VAS score (2.7 ± 1.2), the hospitalization time (7.8 ± 2.8 days), and the Lysholm score at the last follow-up (85.8 ± 5.2). The recurrence rate is significantly lower in groups A (3.1%) and B (5.2%) than group C (40%) (*P* < 0.001).

**Conclusions:**

Arthroscopic treatment for popliteal cysts exhibited better clinical outcomes with minimal invasion and can be recommended for future clinical interventions.

## Background

Popliteal cyst is a common knee joint disease and often seen in elderly patients with knee osteoarthritis or meniscus tear [1, 2]. Traditionally, treatment usually involves open excision from the posterior side of the knee. However, it requires a large incision and is associated with high recurrence rates [3, 4]. It is becoming a commonplace that understanding pathological progression underlying popliteal cysts is beneficial for the current treatment [5]. In recent years, minimally invasive arthroscopy has provided surgeons an alternative approach with prominent advantages [6, 7]. However, arthroscopic treatment alone may not be enough to address both the underlying pathology in the knee joint and the cyst [8]. On the other hand, the combination of arthroscopic treatment and open excision was rarely reported. It is difficult to suggest the best treatment for popliteal cysts because the direct comparison between different surgical approaches with long-term follow-ups are lacking. Here, we sought to compare three different surgical treatments of popliteal cyst: arthroscopic internal drainage, open excision after arthroscopic treatment, and open excision.

## Methods

### Ethics, consent, and permissions

This study was approved by the Ethics Committee of Tai’an Central Hospital. Written information consent to participate was obtained from all patients.

### Patients

A total of 76 patients (35 males and 41 females, age 55.0 ± 8.8 years) with popliteal cysts were enrolled at Tai’an Central Hospital between April 2013 and February 2017. Preoperative magnetic resonance imaging (MRI) and X-ray radiography were conducted to confirm the diagnosis and classify the patients. All patients were scored according to Rauschning and Lindgren classification (RLC) and Kellgren-Lawrence system (K-L) [9]. Patients with K-L grade greater than III, patients with ligament injuries, and patients with recurrent popliteal cysts were excluded in the study. The patients were randomly assigned into three surgical groups using complete block design. Randomly generated numbers were assigned to each patient and divided by 3: remainder 1 was defined as group A, remainder 2 was defined as group B, and remainder 0 was defined as group C. Group A included 32 patients who received arthroscopic internal drainage of the cysts (15 males and 17 females, age 55.3 ± 9.8 years; RLC 20 grade II and 12 grade III; K-L 7 grade 0, 13 grade I, and 12 grade II; 2 patients had cyst only, 13 combined with meniscus injuries, 7 combined with cartilage injuries, 5 had both meniscus and cartilage injuries, 5 combined with synovitis). Group B included 19 patients who received open excision after arthroscopic treatment (9 males and 10 females, age 55.4 ± 7.6 years; RLC 1 grade I, 11 grade II, and 7 grade III; K-L 3 grade 0, 8 grade I, and 8 grade II; 7 patients combined with meniscus injuries, 5 combined with cartilage injuries, 6 had both meniscus and cartilage injuries, 1 combined with synovitis). Group C included 25 patients who received open excision of the cysts (11 males and 14 females, age 54.2 ± 8.5 years; RLC 1 grade I, 13 grade II, and 11 grade III; K-L 4 grade 0, 10 grade I, and 11 grade II; 1 patient had cyst only, 10 patients combined with meniscus injuries, 7 combined with cartilage injuries, 5 had both meniscus and cartilage injuries, 2 combined with synovitis). All procedures were performed by the same surgeon.

### Surgical methods

#### Arthroscopic internal drainage (group A)

**Fig. 1:**
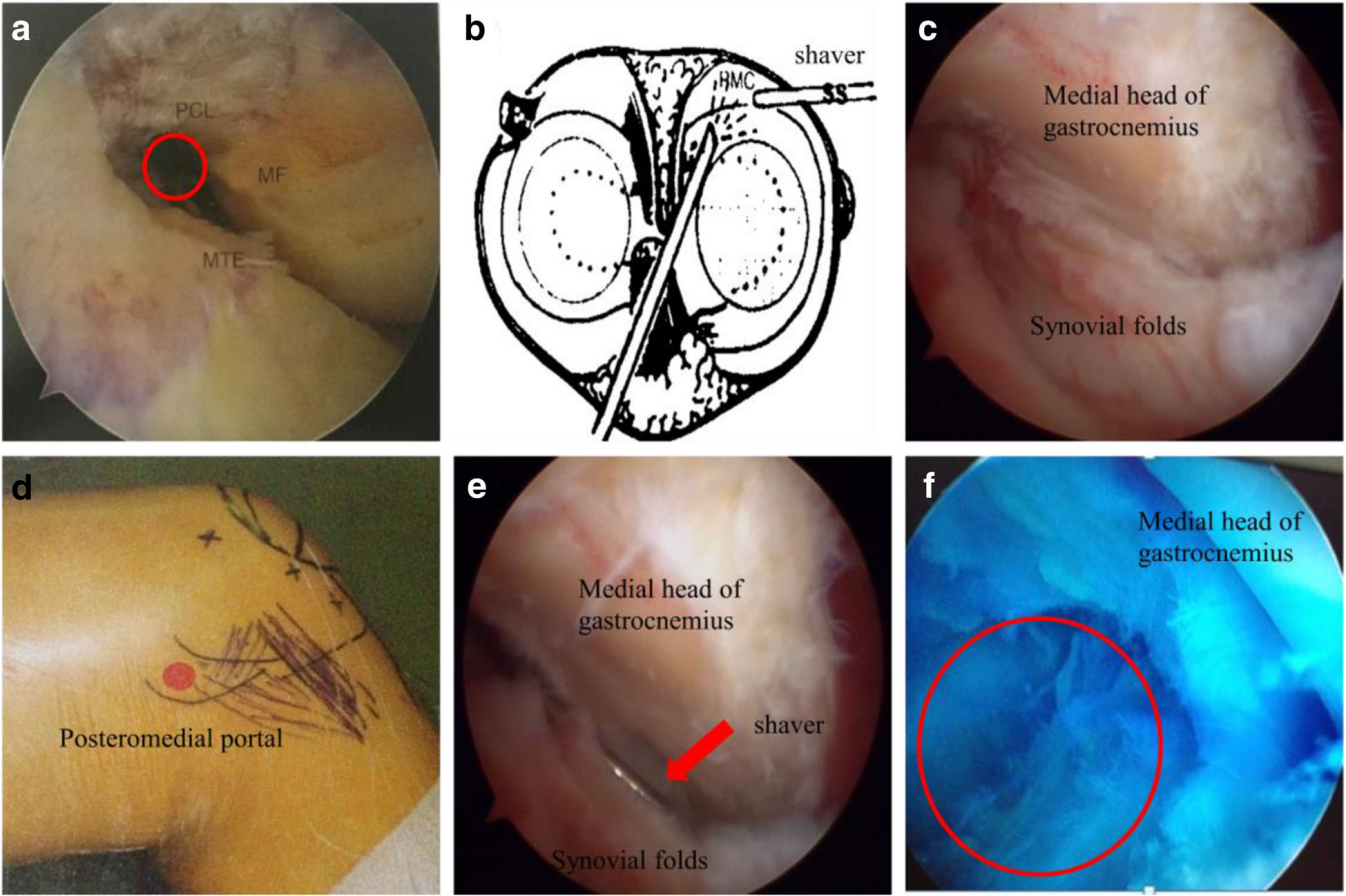
Graphic views of the surgery process of arthroscopic internal drainage. **a** The anterolateral portal where the arthroscope enter (red circle). **b** A schematic view showing the positions of the arthroscopy via anterolateral portal and the shave via posteromedial portal. **c** The arthroscopic view of the medial head of the gastrocnemius muscle and the synovial folds. **d** The posteromedial portal (red dot). **e** The shaver was positioned next to the medial head of gastrocnemius muscle (red arrow). **f** An opening of at least 5 mm in diameter resected by the shaver (red circle). Abbreviations: MF, medial femoral condyle; MTE, medial tibial eminence; PCL, posterior cruciate ligament; PMC, posteromedial corner

Patients were placed in supine position with routine anesthesia or epidural anesthesia. Tourniquet was applied to prevent bleeding during the procedure. However, in order to avoid deep vein thrombosis, tourniquet had to be removed after 1 h and re-applied 10 min later. The procedure was paused during the 10 min. If bleeding occurred, plasma knife was used to stop bleeding. After disinfection, 1–2 ml methylene blue was injected into the cyst to identify the valvular opening. An arthroscope was used to detect any meniscus or cartilage injuries and synovial hyperplasia. Next, an arthroscope was inserted through the anterolateral portal into the posteromedial compartment, via the space between the posterior cruciate ligament and the medial femoral condyle (Fig. 1a, b). Posteromedial compartments were visualized to confirm the position of medial gastrocnemius tendon and the transverse posteromedial synovial folds and the opening of the cyst (Fig. 1c, d). The posteromedial portal was then established under the light, and a shaver was inserted and placed next to the medial head of the gastrocnemius muscle to clean the joint space and the opening of the cyst (Fig. 1e). At this time, outflow of cyst fluid was visualized by methylene blue, and the opening was incised to at least 5 × 5 mm (Fig. 1f). If there existed meniscus or cartilage injuries, appropriate repair surgeries were performed immediately. A total of 18 patients received meniscus repairs and 12 received cartilage repairs. Finally, the joint capsule was thoroughly cleaned and flushed after hemostasis. Limb restraint was not necessary after the procedure. Quadriceps exercises started as early as 6 h postoperative time. Elastic bandages and ice cubes were applied at the surgical site and were removed after 2–3 days. Patients could start to walk after 3 days and were discharged after 5 to 7 days.

#### Open excision after arthroscopic treatment (group B)

Patients were placed in supine position with routine anesthesia or epidural anesthesia. An arthroscope was utilized to clean the joint space and check for any meniscus or cartilage injuries and synovial hyperplasia. A total of 13 patients received meniscus repairs, and 11 received cartilage repairs. Next, patients were switched to the prone position for open excision. An S-shaped incision (8 to 12 cm) was made in the medial popliteal area. The deep fascia was incised longitudinally to expose the cyst. Blunt dissection was used around the cyst close to the semimembranosus muscle, the medial gastrocnemius muscle, and the bursa. The cyst was kept intact to avoid vessel and nerve damage. The cyst was lifted and cut off at the root. If the cyst was connected to the joint space, suturing was performed. Thoroughly flushing and suturing were the final steps. Bandage with moderate pressure and a drainage tube were placed after the procedure. The knee was restrained in the extension position. Quadriceps exercises started the next day. Patients were able to walk with cane after 7 days when post-surgical pain was largely relieved and subject to knee exercises strengthening gradually. Sutures were removed after 12–14 days.

#### Open excisions (group C)

Open excisions were performed in the same way as described in group B.

### Follow-up visits

All functional evaluations during follow-up visits were performed by independent clinicians who were blinded to the treatment. Incision healing was assessed repeatedly on days 1, 2, 3, 5, 7, and 12 post surgery. The incision healing was evaluated as follows: grade 1, good healing with no adverse events; grade 2, redness, induration, and hematoma or fluid build-up around the incision; grade 3, suppuration around the incision that requires surgical drainage. The postoperative pain visual analog scale (VAS) score was assessed on the third day after surgeries. At the last follow-up visits, all patients underwent MRI scanning. The popliteal cysts were evaluated according to RLC, and knee functions were evaluated by Lysholm scores.

### Statistics

Statistical analysis was performed using SPSS 18.0. The data were presented as mean ± standard deviation. Comparisons were done by one-way ANOVA with least significant difference post hoc test or chi-square test with chi-square partitioning. An *α* of 0.05 is considered as the cutoff for statistical significance.

## Results

There was no significant difference in preoperative measurements among the three groups of patients, including age, gender, healing rate, preoperative Lysholm, and pain VAS scores (Table 1). The three groups were also no comparable in perioperative complications such as popliteal hematoma, neural or vascular injury, and symptomatic deep vein thrombosis (DVT) or pulmonary embolism (PE), as well as follow-up time. During arthroscopic treatment, those patients with co-existing injuries received repairing surgeries for management perspective. In total, 18 patients had meniscal surgery and 12 had chondral surgery in group A, whereas 13 patients had meniscal surgery and 11 had chondral surgery in group B.

**Table 1:** Patient information

		Group A	Group B	Group C	*F*/*x* ^2^	*P* value
Age		55.3 ± 9.8	55.4 ± 7.6	54.2 ± 8.5	0.151	0.860
Gender	Male	15	9	11	0.064	0.968
	Female	17	10	14		
Preoperative Lysholm score		47.3 ± 5.7	47.3 ± 5.4	47.4 ± 4.7	0.001	0.999
Preoperative VAS score		6.5 ± 0.8	6.6 ± 0.9	6.2 ± 0.7	1.769	0.178
Perioperative complications		0	0	0		
Symptomatic DVT/PE	Yes	4	3	4	0.174	0.917
	No	28	16	21		
Follow-up time (months)		13.8 ± 2.7	13.7 ± 2.2	13.7 ± 2.3	0.006	0.994

Patients in group A did significantly better in terms of incision healing (100% grade 1), the pain VAS score 3 days after surgery (2.7 ± 1.2), the total hospitalization time (7.8 ± 2.8 days), and the Lysholm knee scaling score at the last follow-up (85.8 ± 5.2) compared to group B and group C (Table 2; *P* < 0.05). There was no significant difference between group B and group C (Table 3). Group A also had significantly shorter incision (1.6 ± 0.1 cm) than group C (10.6 ± 1.8), whereas group C was significantly shorter than group B (13.7 ± 2.7). As expected, group B required significantly longer operative duration (109.2 ± 25.4 min) compared to both group A (63.7 ± 12.7 min) and group C (62.5 ± 9.6 min).

**Table 2:** Surgery and post-surgery data

		Group A	Group B	Group C
Surgical time (min)		63.7 ± 12.7	109.2 ± 25.4	62.5 ± 9.6
Length of incision (cm)		1.6 ± 0. 1	13.7 ± 2.7	10.6 ± 1.8
Incision healing	Grade 1	32	14	18
	Grade 2	0	5	7
Postoperative VAS score		2.7 ± 1.2	4.1 ± 0.7	3.8 ± 0.8
Hospitalization time (days)		7.8 ± 2.8	15.9 ± 3.4	15.9 ± 5.5
Recovery to grade 0 or I cysts		96.9%	94.8%	60%
Recurrence rate		3.1%	5.2%	40%
Lysholm score at last follow-up		85.8 ± 5.2	80.3 ± 3.9	78.9 ± 5.0

**Table 3:** Surgery and post-surgery data (post hoc analysis)

	Group A vs B		Group A vs C		Group B vs C	
	*t*/*x* ^2^	*P* value	*t*/*x* ^2^	*P* value	*t*/*x* ^2^	*P* value
Surgical time (min)	− 45.523	< 0.001	1.208	0.779	46.731	< 0.001
Length of incision (cm)	− 12.109	< 0.001	− 9.012	< 0.001	3.097	< 0.001
Incision healing rate	9.336	0.002	10.214	0.001	0.015	0.901
Postoperative VAS score	− 1.449	< 0.001	− 1.104	< 0.001	0.345	0.228
Hospitalization time (days)	− 8.051	< 0.001	− 8.076	< 0.001	− 0.025	0.983
Recovery to grade 0 or I cysts	0.145	0.704	12.254	< 0.001	6.947	0.008
Recurrence rate	0.145	0.704	12.254	< 0.001	6.947	0.008
Lysholm score at last follow-up	5.581	< 0.001	6.964	< 0.001	1.383	0.349

**Fig. 2:**
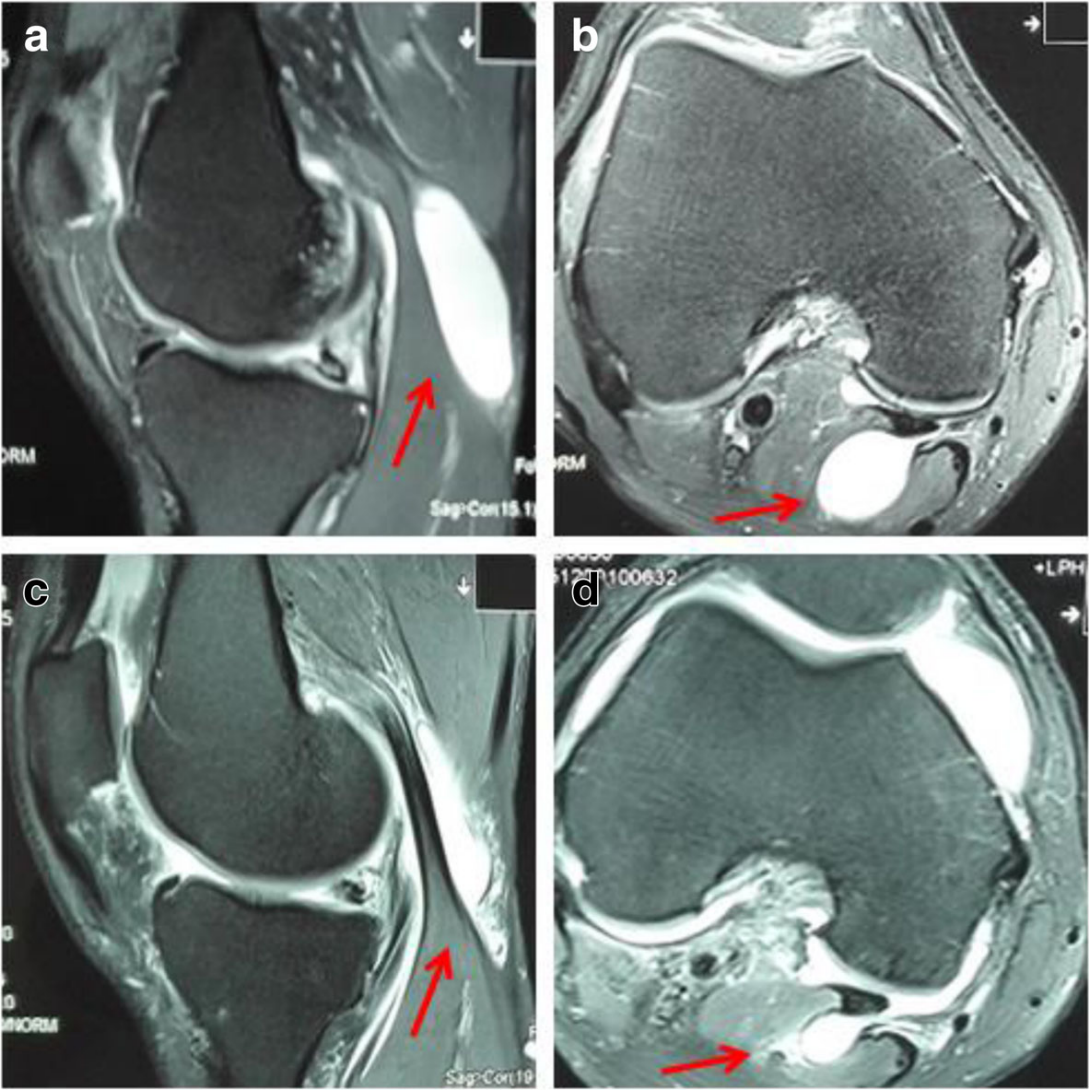
Representative MRI images from group A patient, after receiving arthroscopic internal drainage. **a**, **b** Preoperative sagittal and axial views around the knee joint. **c**, **d** Postoperative sagittal and axial images at 2 months follow-up exhibited substantial shrinkage of the cyst. Red arrows indicate the popliteal cyst

In group A, we observed that the cysts started to shrink 2 months post surgery in 13 patients (Fig. 2). In 8 patients, the cysts completely disappeared by 8 months under MRI. At the last follow-up visits, which were on average 13.7 months after the surgery, an independent clinician who was blinded to the treatment scored all patients according to the Rauschning and Lindgren classification. The patients in grade II and above were defined as recurrent patients. The recurrence rates were significantly lower in group A (3.1%) and group B (5.2%) than those in group C (40%).

## Discussion

Popliteal cysts, also called Baker’s cysts, are often seen at the gastrocnemius-semimembranosus bursa behind the knee joint [10]. Although the exact mechanisms for popliteal cysts are not clear, it is widely accepted that they result from a one-directional flow of knee effusion into the bursa through a valvular opening [11, 12]. Most popliteal cysts are connected to the knee joint space and often associated with intra-articular pathology [11–13]. Traditional treatment for popliteal cysts is using open excision from a posterior incision. However, this surgery usually involves extensive exposures and risk of neural or vascular injuries. Patients are prone to scar formation after surgery, resulting in unsatisfying cosmetic appearance [14]. More importantly, open excision does not address the associated intra-articular pathology and has a high recurrence rate [4, 15]. Based on the one-way valve mechanism, we believe that the key to successful treatment of popliteal cysts is dealing with the associated intra-articular lesions and re-establishing the bi-directional communication between the bursa and the joint space [16]. Arthroscopic internal drainage for popliteal cysts has become widely accepted in the recent years [6, 7].

In this study, we compared three different surgical approaches for popliteal cysts: arthroscopic internal drainage, open excision after arthroscopic treatment, and open excision alone. Arthroscopic internal drainage and open excision required less surgical time. In four patients of group A, arthroscopic internal drainage can be achieved within 45 min. On the other hand, the combination treatment needed much longer time, among which two surgeries lasted more than 2.5 h. Arthroscopic internal drainage was significantly better than the other approaches in the following parameters: the incision length, the incision healing rate, VAS pain score after surgery, and the Lysholm score at the last follow-up. Importantly, more than 1 year after the surgeries, we only observed one case of recurring cysts in patients receiving arthroscopic or combination treatment respectively. The recurrence rate was 3.1% for arthroscopic internal drainage and 5.2% for combination treatment, compared to 40% for open excision. It was consistent with previous publications that open excision procedure without other interventions leads to a higher recurrence rate [4, 9].

Arthroscopic treatment requires the smallest incision and is more effective to improve knee functions. It is capable of cleaning lesions inside the knee joint while simultaneously re-establishing the bi-directional communication between the cyst and the joint capsule [10]. Additionally, arthroscopic treatment is more cost-efficient by significantly shortening the hospitalization time.

Arthroscopic approach requires sufficient sterile saline, which can flush out free radicals and inflammatory cytokines, leading to reduced infection, inflammatory responses, and pain postoperatively. We did not encounter infection in the arthroscopic internal drainage group. In the combination group, patients needed to be turned over and disinfected again, resulting in much longer procedure time, with increased the risk of infection [17]. Therefore, although very good outcomes of the combination treatment were observed here and others [18], the shorter operation duration and low infection rates favored arthroscopic internal drainage. Additionally, because of less pain scores in arthroscopic internal drainage, patients were able to walk earlier, which effectively prevented vein thrombosis in lower limbs. This resulted in shorter hospitalization time, which was cost-efficient.

## Conclusions

Our results suggest that it is critical to clear intra-articular pathology for the successful treatment of popliteal cyst, whereas the removal of the cyst is not the primary goal for surgical intervention. Arthroscopic internal drainage of the cyst not only treats the intra-articular lesions but also re-establishes the bi-directional communication between the bursa and the joint space. The minimally invasive procedure has small incision, quick recovery, good efficacy, and low recurrence rate. Although it may require a longer learning curve for the technique, we would recommend arthroscopic treatment for popliteal cysts in clinical practice.

## Abbreviations

DVT: Deep vein thrombosis
PE: Pulmonary embolism
VAS: Visual analog score

## References

[1] Katz JN, Brownlee SA, Jones MH (2014). The role of arthroscopy in the management of knee osteoarthritis. Best Pract Res Clin Rheumatol.

[2] Labropoulos N, Shifrin DA, Paxinos O (2004). New insights into the development of popliteal cysts. Br J Surg.

[3] Takahashi M, Nagano A (2005). Arthroscopic treatment of popliteal cyst and visualization of its cavity through the posterior portal of the knee. Arthroscopy.

[4] Rupp S, Seil R, Jochum P (2001). Long-term results after excision of a popliteal cyst. Unfallchirurg.

[5] Malinowski K, Synder M, Sibinski M (2011). Selected cases of arthroscopic treatment of popliteal cyst with associated intra-articular knee disorders primary report. Ortop Traumatol Rehabil.

[6] Lie CW, Ng TP (2011). Arthroscopic treatment of popliteal cyst. Hong Kong Med J.

[7] Ohishi T (2015). Treatment of popliteal cysts via arthroscopic enlargement of unidirectional valvular slits. Mod Rheumatol.

[8] Rupp S (2002). Popliteal cysts in adults. Prevalence, associated intraarticular lesions, and results after arthroscopic treatment. Am J Sports Med.

[9] Rauschning W, Lindgren PG (1979). Popliteal cysts (Baker’s cysts) in adults. I. Clinical and roentgenological results of operative excision. Acta Orthop Scand.

[10] Kim KI (2014). Arthroscopic anatomic study of posteromedial joint capsule in knee joint associated with popliteal cyst. Arch Orthop Trauma Surg.

[11] Ko S, Ahn J (2004). Popliteal cystoscopic excisional debridement and removal of capsular fold of valvular mechanism of large recurrent popliteal cyst. Arthroscopy.

[12] Kim TW, Suh JT, Son SM, Moon TY, Lee IS, Choi KU, Kim JI (2012). Baker’s cyst with intramuscular extension into vastus medialis muscle. Knee Surg Relat Res.

[13] Sansone V (1995). Popliteal cysts and associated disorders of the knee. Critical review with MR imaging. Int Orthop.

[14] Bohensky MA (2014). Quantifying the excess cost and resource utilisation for patients with complications associated with elective knee arthroscopy: a retrospective cohort study. Knee.

[15] Fritschy D (2006). The popliteal cyst. Knee Surg Sports Traumatol Arthrosc.

[16] Sansone V (2004). An unusual cause of popliteal cyst. Arthroscopy.

[17] Pinkowsky GJ, Lynch S (2013). Locked knee caused by lateral meniscal capsular disruption: verification by magnetic resonance imaging and arthroscopy. Am J Orthop (Belle Mead NJ).

[18] Saylik MGK (2016). Treatment of baker cyst, by using open posterior cystectomy and supine arthroscopy on recalcitrant cases (103 knees). BMC Musculoskelet Disord.

